# Physiological Synchrony of the Broad Bean Weevil, *Bruchus rufimanus* Boh., to the Host Plant Phenology, *Vicia faba* L.

**DOI:** 10.3389/finsc.2021.707323

**Published:** 2021-08-06

**Authors:** Rachid Hamidi, Pierre Taupin, Brigitte Frérot

**Affiliations:** ^1^INRAE, UMR 1392, Institute of Ecology and Environmental Sciences of Paris (IEES Paris), Versailles Cedex, France; ^2^ARVALIS, Institut du Végétal, Boigneville, France

**Keywords:** Bruchids, spermatheca, oogenesis, IPM, crop protection, pollen, insect life cycle

## Abstract

The aims of the study were to understand the physiological and phenological relationship between *Bruchus rufimanus* and *Vicia faba* in the perspective of IPM strategies. *V. faba*, an important food resource for humans and livestock is the main host plant of *B. rufimanus*. Adults feed on the pollen, females lay eggs on pods, and larvae develop into the seeds. Pending the blooming phase, the weevil may feed on the pollen from wild plants. Depending on the sowing date, the phenological time lag should lead the plant parts, most utilized by the weevil, less or more available during the key life stages of the pest. The aims of the study were therefore (1) to assess the impact of the sowing date (i.e., cultivars) on the phenological relationship between *B. rufimanus* and *V. faba*, and (2) to identify alternative pollen resources during the vegetative phase. Insects were collected weekly on two cultivars: winter-sown and spring-sown fields. Ovarian development, fecundity, and diet were monitored using dissected adults. Results showed that immature adults colonized the blooming winter-sown field and then 2 weeks later, the blooming spring-sown field. Sexual maturity of the weevils is related with *V. faba* pollen consumption. The sexual maturity of females increased with the growing density of flowers and the first pods were quickly covered with eggs. In spring-sown field, first pods grew 19 days later, while collected females in winter sown field had already laid most of their eggs. Feeding tests were carried using flowers collected from plants growing close to the fields: wild chervil, oilseed rape and wild Prunus. All this pollen were consumed by the weevils. The study showed a perfect synchrony between the host and the pest. Late sowing date and control of the early blooming wild plants surrounding the crops might reduce the attacks in field beans. More broadly, the study helps to understand the eco-ethology of the insect in cultivated areas.

## Introduction

Seed beetles developing on a single species or on some closely related ones, rely on the plant lifespan and phenology, and have developed a close relationship with the host plant. Indeed, throughout co-evolution, plants have strongly influenced the life cycles of monophagous insects. If some relationships between insects and plants prove mutually beneficial, such as pollination, most of them involve plant consumption, and therefore plant responses (or plant defenses). In bruchid beetles, the species have specialized in Fabaceae and they have co-evolved (1). Blooming and fruit set up periods strongly reflect the evolutionary compromises in response to the weevil's presence (2, 3). Typically, adults first feed on the plant pollen, then females lay eggs on pods, and finally, larvae feed and develop into the seeds. Therefore, in Fabaceae, the plant phenology, such as blooming and pod setting, are components of the selective pressure driving the optimal physiological development of bruchid beetles (4–7).

*Vicia faba*, also named field beans or broad beans, is the most common crop belonging to Fabaceae. *V. faba* is native to the Mediterranean region, where it has spread through farming to the rest of the world (8). *V. faba* is an important food resource for humans and livestock. China is the largest producer with 1.80 million tons (2017), followed by Ethiopia, Australia, and the United Kingdom. Germany and France complete the top six with 188 thousand tons (9). The recent popularity of broad beans is due to numerous facts, such as studies demonstrating that *V. faba* is a healthy food and brings environmental and agricultural benefits (10, 11). In France, the economically accepted damage caused on the seed, must not exceed 3% for human consumption and 10% for animal products (12). Seeds can be sown in winter (i.e., the winter-sown field) and in spring (i.e., the spring-sown field) (13). Winter-sown broad beans bloom earlier than spring-sown broad beans.

*Bruchus rufimanus* is common in Palearctic region and is monophagous developing mainly on *V. faba*. This Coleoptera, belonging to the Chrysomelidae family, Bruchinae subfamily, is also called seed beetle, broad bean weevil and bruchid beetle. *B. rufimanus* is one of the most serious pests on *V. faba*, and is poorly controlled by the remaining permitted conventional insecticides. The level of damage tends to reduce exportations and farmers' incomes. The problem dates back a long way. There is archaeological evidence that the seeds for human consumption have already been infested by *B. rufimanus* (14). Unlike other bruchid weevils, *B. rufimanus*, does not develop as a stored product pest, but overwinter as adults around fields and hedges, and under tree barks and lichens (5, 15). *B rufimanus* is univoltine and the reproductive diapause is compulsory. Photoperiod and temperature conditions regulate the end of the diapause (4). In spring, when temperatures reach daily maxima, from 17 to 20°C, broad bean weevils leave overwintering sites and migrate to *V. faba* fields, where they feed on pollen (15, 16). When spring emergence occurs, *V. faba* is in a vegetative phase, bearing young leaves. Therefore, pollens from alternative plant species remain a vital resource for the pest. Little is known about alternative pollen resources; the present paper aims to clarify this point, especially the role of wild chervil (*Anthriscus sylvestris* L., Apiaceae), the wild Prunus (*Prunus* sp., Rosaceae), and oilseed rape (*Brassica napus* L., Brassicaceae), all blooming earlier and growing close to overwintering sites or crops.

Related to the trend of decreasing insecticide treatments, several strategies are used to reduce the damage caused by the broad bean weevil to more acceptable levels (17). Strategies can include making the environment less suitable for the pest survival. For example, (1) changing the sowing date (i.e., cultivars) can make the plant resources that are mostly utilized by the weevil (i.e., flowers and pods), less/more available during the key life stages of the pest. (2) Reducing alternative pollen resources during the vegetative phase of broad beans, or during crop rotations, should negatively impact local populations. The specific host plant recognition and infestation of different cultivars of *V. faba* address several questions: In which way plant phenology influences the broad bean weevil infestation, and therefore, how the insect physiology is synchronized to the host plant phenology. Are there alternative pollen resources to maintain post overwintering populations before the *V. faba* blooming phase?

To that end, insect physiology was studied by dissecting insects collected in winter-sown and spring-sown fields. Pollen feeding tests were carried out, as well as determining which pollen was ingested. Results were discussed in light of the eco-ethology knowledge of the pest.

## Materials and Methods

### The Plants

The study was conducted at ARVALIS experimental station (Boigneville, France) (48°32 N, 2°38 E). Two cultivars of broad bean, belonging to the variety group of “minor,” were used: the commercial field bean cultivar sown in winter, referenced as “DIVA,” and the spring-sown broad bean commercial cultivar, referenced as “ESPRESSO.” Both cultivars were sown on separate plots, sizing 24 × 36 m each, and one meter apart. The fields were surrounded by crops, such as pea (*Pisum sativum* L., Fabaceae), rapeseed (*Brassica napus* L., Brassicaceae), and some wild plants, such as wild chervils (*Anthriscus sylvestris* L., apiaceae) and wild Prunus (*Prunus* sp., Rosaceae). No pesticides, chemical fertilizers, or herbicides were sprayed on fields.

### Monitoring the Phenology of Broad Bean Fields

From April 14 to July 5, 2011, at least once per week, 20 plants were observed to assess the phenology of the cultivars. The phenology was assessed according to ARVALIS code, using the number of nodes per stem bearing inflorescences, and the number of nodes per stem bearing pods. Then, the modal value of the phenology was used.

### Monitoring Broad Bean Weevils in the Fields

**Figure 1:**
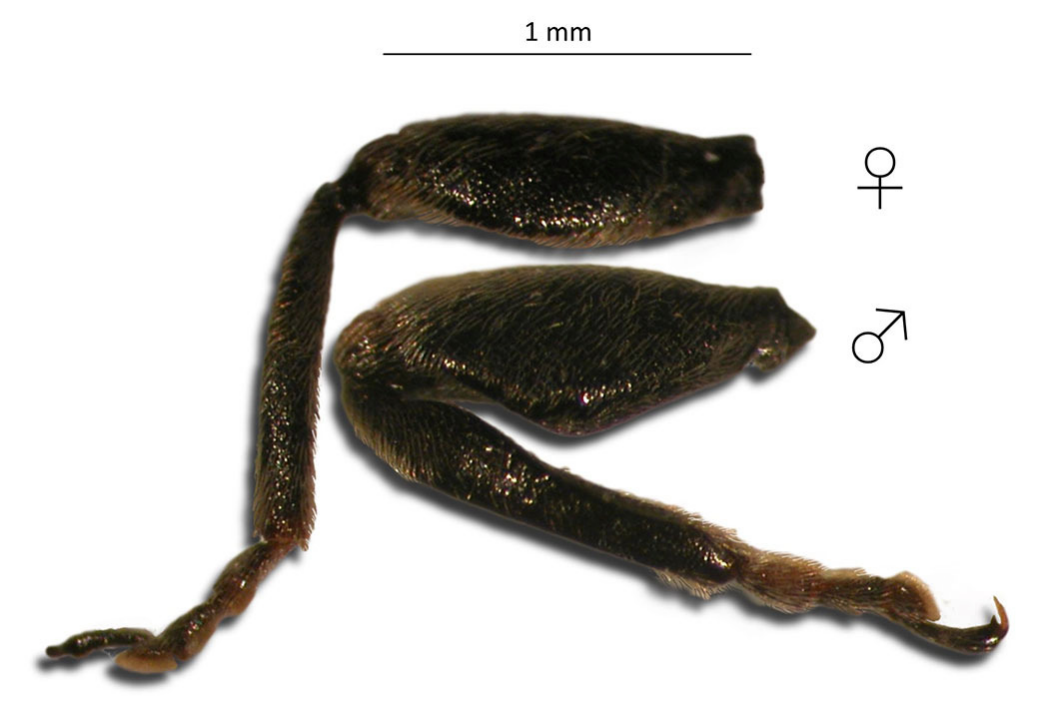
Right middle legs of a male and a female (X20 magnification).

To determine the relationship between *V. faba* phenology and populations of broad bean weevils, in both cultivars, and at each phenological observation (as previously described), broad bean weevils were collected from 100 randomly chosen plants. Insects were collected in the morning when the temperature was cooler and insects were relatively motionless. Broad bean weevils were counted, and then sexed according to middle-leg dimorphisms between males and females (Figure 1) (18).

**Figure 2:**
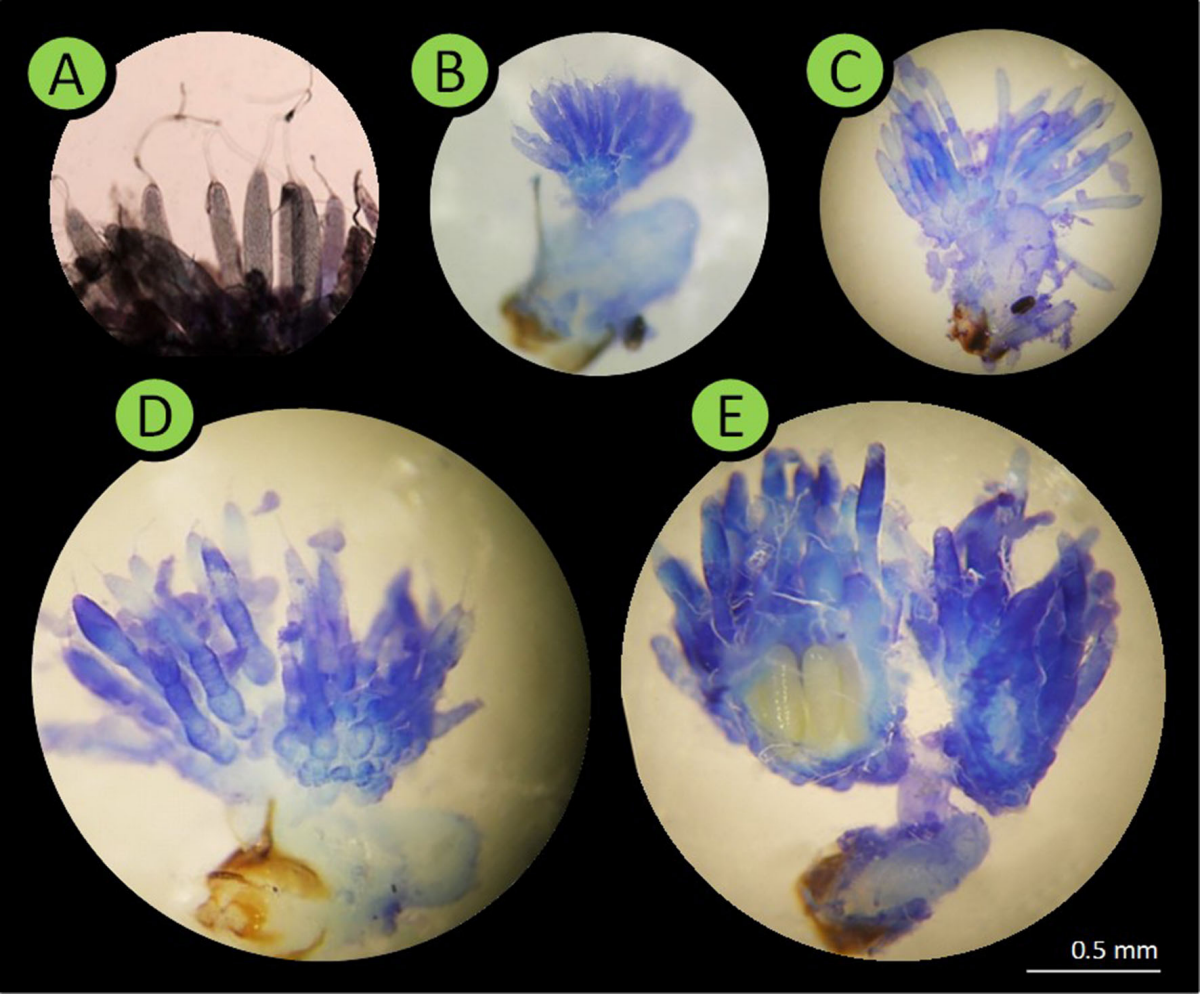
Pictures of *Bruchus rufimanus* female reproductive system (X20 magnification). **(A,B)** Rank 0, female in reproductive diapause, the ovarioles were reduced to their *germarium* part. **(C,D)** Ranks 1–3, the ovarioles were longer, the *germarium* was reduced and the oocytes development was clear. **(E)** Rank 4, at least one oocyte was present in the lateral oviducts.

**Figure 3:**
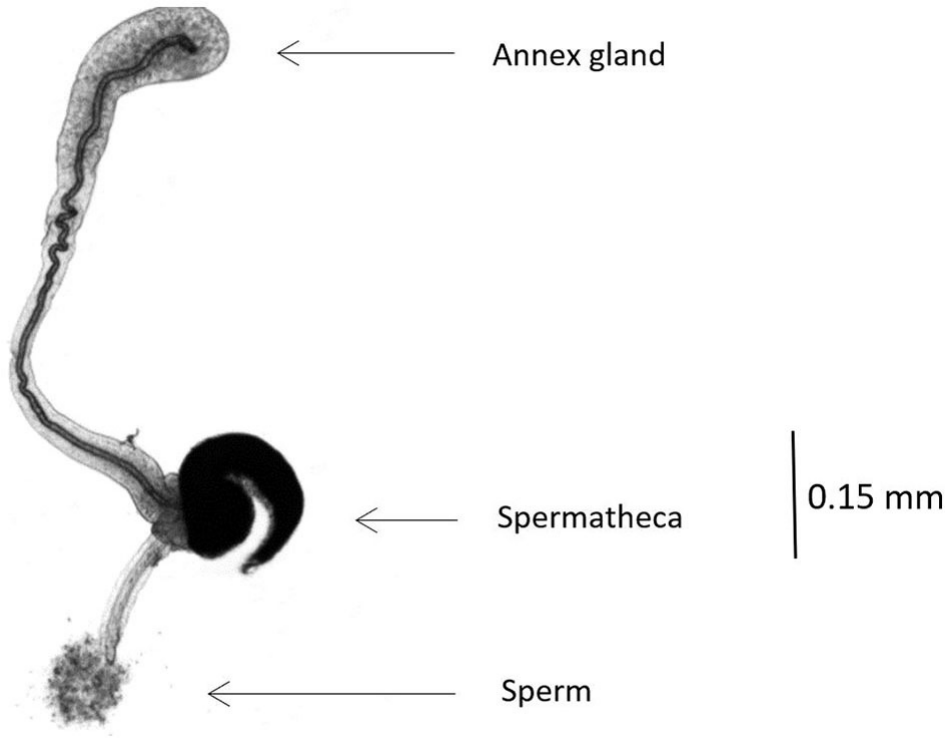
Picture of full spermatheca in *B. rufimanus* (X50 magnification).

In winter-sown and in spring-sown field beans, 7.76 ± 3.74 and 4.94 ± 1.76 (mean ± SD) insects were weekly dissected, respectively. Their legs, elytra and wings were removed prior to dissection. The insect was pinned ventral side-down in a Petri dish with solid paraffin at the bottom. A physiological solution was used for all dissections and measurements. A stereo microscope was used for observing spermatozoids in the spermatheca. In males, sexual maturity development was assessed by comparing the size of the lateral gland and the median gland of diapausing males. Sexually mature males had enlarged glands (19). Respectively, 28 and 12 males collected from winter-sown field beans (from April 28 to June 28) and spring-sown broad beans (from May 6 to June 30), were dissected. In females, sexual maturity was assessed using the rank of ovarian development and spermatheca filled with spermatozoids (Figure 2). To calibrate the rank, five females just emerged from seeds were dissected. In diapausing females, the ovarioles were reduced to the *germarium* (described as rank 0), whereas, in sexually mature females, mature oocytes were present and ovarioles were fully developed (described as rank 4). Forty-seven females from winter-sown broad beans (from April 28 to June 28), and 23 females from spring-sown broad beans (from May 6 to June 30) were dissected. The mating percentage was assessed using the number of females with a spermatheca filled with spermatozoids (Figure 3). To assess the ovarian development rank and the presence of filled spermatheca, 44 females collected from winter-sown broad beans (from April 28 to June 28), and 22 females collected from spring-sown broad beans (from May 6 to June 30), were dissected.

Diet was assessed through dissections of midguts and hindguts of males (*n* = 28) and females (*n* = 47) that were collected from winter-sown broad beans (from April 28 to June 28), and males (*n* = 12) and females (*n* = 23) collected from spring-sown broad beans (from May 6 to June 30). Pollen grains were identified to assess which plants insects had fed on.

To quantify the body fat quantity, different ranks were established. The highest rank corresponded to insects freshly hatched from the seeds, and the lowest rank to insects collected from the fields at the end of the capture season. For the highest rank, body fat filled the entire abdomen, whereas for the lowest rank, almost no body fat was left. From the winter-sown broad beans, 28 males and 47 females were dissected; and from the spring-sown broad beans, 9 males and 23 females were dissected (X20 magnification).

### Nutrition

To test their ability to feed on pollen from alternative plant species, three couples of broad bean weevils were collected from the winter-sown broad beans, and kept with inflorescences from alternative plant species: wild chervil (*Anthriscus sylvestris*), oilseed rape (*Brassica napus*), or wild Prunus (*Prunus* sp.). In addition, three couples were kept with inflorescence from *V. faba* as a control test. The couples were kept under the following conditions: constant temperature at 25°C, 35% r.h., and 18L:6D photoperiod produced by full-spectrum light lamps (TrueLight^©^). After 1 week, pollen consumption was tested by the observation of pollen residues in the midgut, hindgut and feces (X50 magnification).

### Climatic Parameters

In the field, insects were highly impacted by climatic conditions, this was notably the case for the following behaviors: leaving overwintering sites to crops, moving between plant organs, or even ovipositioning behavior. Therefore, climatic parameters had to be taken into account. Climatic parameters were recorded using a connected weather station (Weenat®), located at the experimental station of Arvalis (Boigneville, France). Maximal temperature (°C) and sunshine hours were recorded during the study.

### Statistical Analyses

Mann-Whitney's test was done to compare the mean number of broad bean weevils collected in each cultivar, and the differences in body fat between males and females. Linear regression was used to follow the physiological ranks of weevils during the infestation (i.e., the ovarian development, the number of filled spermatheca, the number of mature oocytes, and the body fat reserve). Correlations between the rate of full mated females and the ovarian development rank were done, using a Spearman test. The test was carried out using GraphPad Instat 3.10 software (GraphPad Software, San Diego California USA). Finally, non-linear curves were fitted to predict the relationship between previous physiological parameters over time. NLFit module in ORIGIN 2016 software (Origin Lab, Northampton, MA) was used.

## Results

### Winter-Sown Broad Beans: Vegetative Phase

From 14 to 24 April 2011, winter-sown fields were in a vegetative phase. No broad bean weevils were observed. Temperatures were between 15 and 25°C.

### Winter-Sown Field: First Week of the Blooming Phase

**Figure 4:**
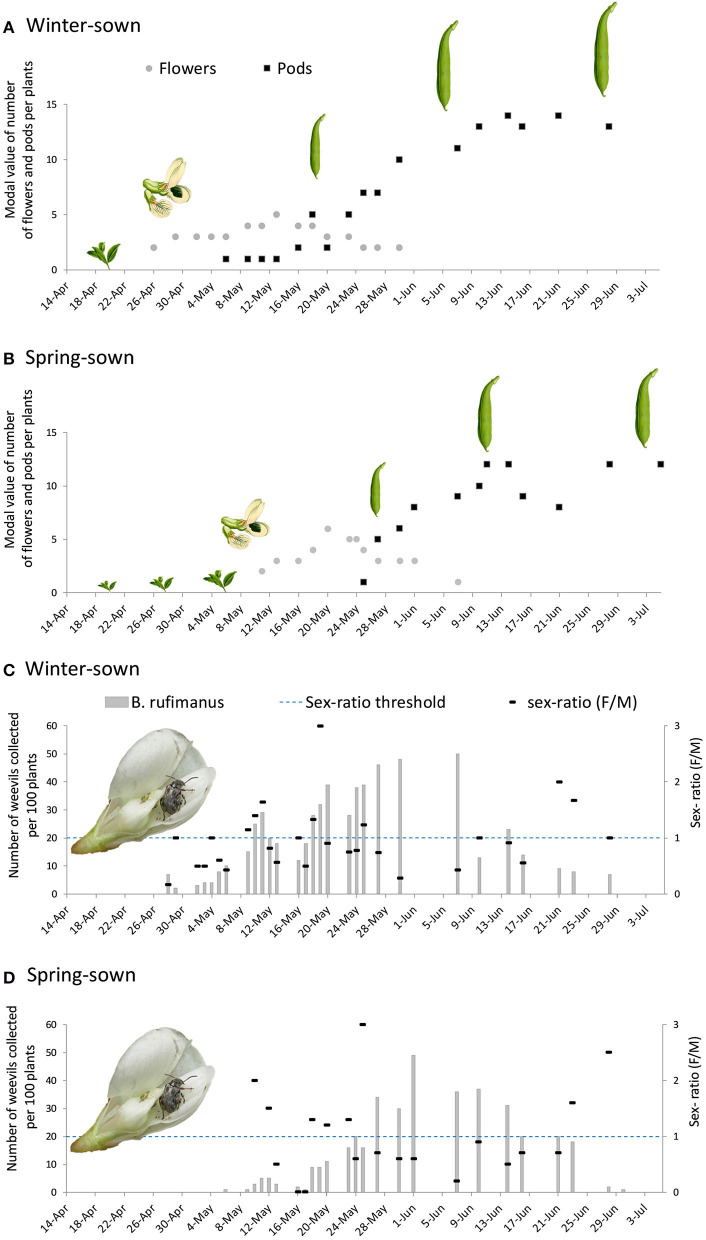
Number of nodes bearing inflorescences and pods in **(A)** winter-sown and **(B)** spring-sown field beans. Number of *B. rufimanus* and sex-ratio over time in **(C)** winter-sown and **(D)** spring-sown field beans.

**Figure 5:**
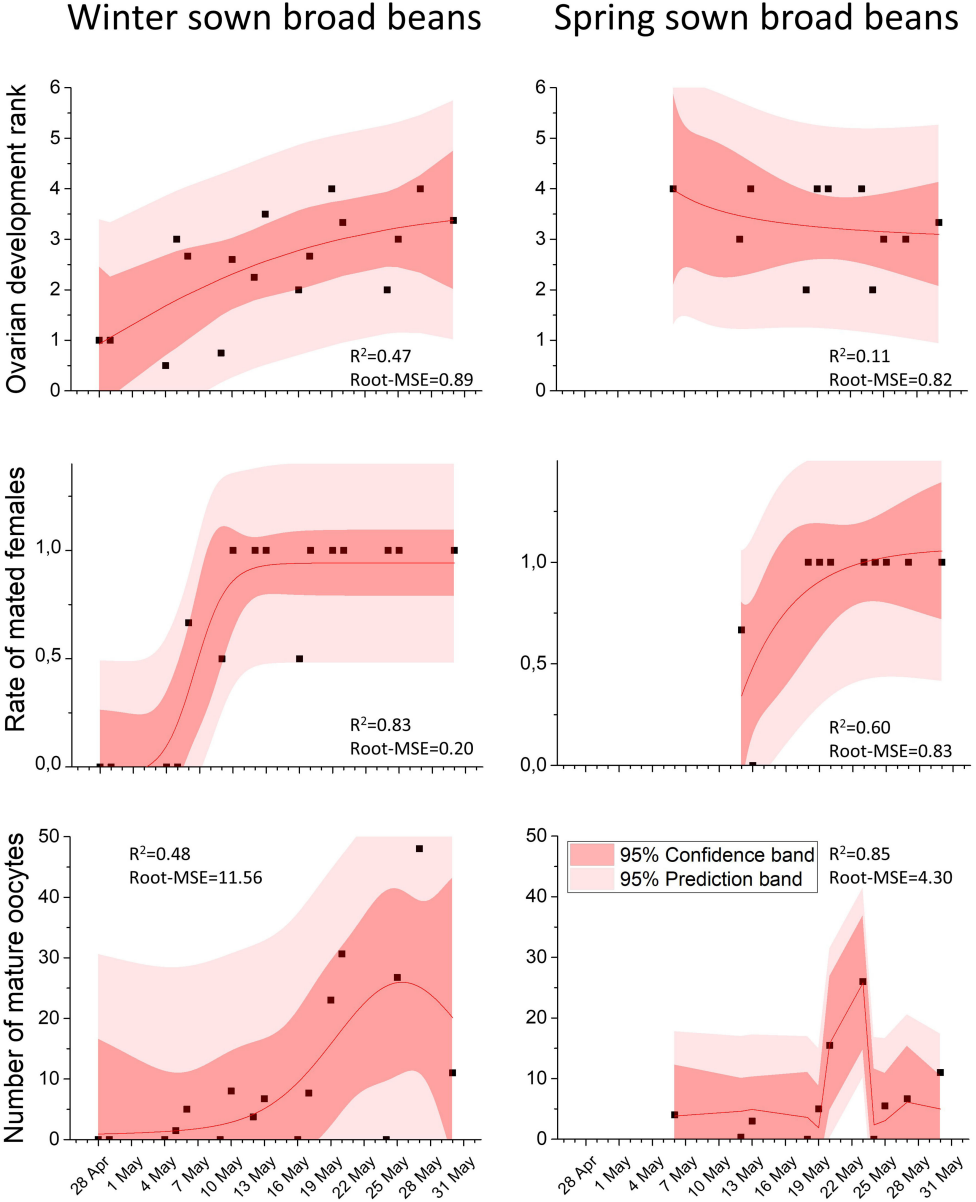
In scatter plot, the mean of female ovarian development rank, the mean of the rate of mated females and the mean of mature oocytes over time in collected females in winter-sown and spring-sown broad beans. The non-linear curve fitting (in red lines), R-square and Root Mean Square Error values were obtained from non-linear curve fitting analysis, using NLFit module from ORIGIN 2016 software.

In winter-sown field beans, flowers were observed from April 26, and the first weevils were collected on April 28 (Figures 4A,C). During the first week of infestation, the sex ratio was temporally male biased. Among dissected males, 37% showed enlarged lateral and median accessory glands. In females, few showed developed ovarioles. The rank observed was between 0.5 and 1.0. All females bore empty spermatheca (Figure 5).

### Winter-Sown Field: Blooming and Fructification Phase

From the end of April, the number of nodes per stem bearing inflorescences increased progressively, peaking at five inflorescence stage on May 13. Throughout the entire period of the weevil presence on the field (from April 28 to June 28), no significant sex ratio bias was observed: 10.79 males (SD ± 8.67) and 9.03 females (SD ± 6.68) (Mann-Whitney test, *p* = 0.50) (Figure 4C). During this period, the ovarian development rank increased (non-linear regression, *R*^2^ = 0.47, R-MSE = 0.89, *n* = 17, Df = 13; Figure 5). The number of ovocytes showed a peak on May 25 (±0,5 days), and then decreased (non-linear regression, *R*^2^ = 0.48, R-MSE = 11.56, *n* = 17, Df = 12; Figure 5). In the broad bean field, mating was regularly observed. Dissections of collected insects showed an increasing number of mated females, following a sigmoid curve. The rate reached a plateau from May 10 (non-linear regression, *R*^2^ = 0.83, R-MSE = 0.20, *n* = 16, Df = 12; Figure 5).

First filled spermatheca matched with the first pods observed on the plant in the field (i.e., May 6, Figure 4A). Then in late May, there was a subsequent decline in the number of inflorescences, but the number of insects was still high (about 10 insects per plant) and all dissected females were mated. The fructification period started from May 6 and first eggs were observed from May 13, while pods were still small. At this stage, the plant had five nodes with inflorescences per stem, and one to two nodes with pod sets per stem. A rapid expansion of pods was observed between May 23 and May 29.

The dynamic of pods number followed a sigmoid curve, which reached a plateau on June 27, with 13.58 ± 0.58 pods (non-linear regression, *R*^2^ = 0.97, R-MSE = 0.96, *n* = 17, Df = 13; Figure 4). At that period, corresponding to the end of colonization and pods presence, sex ratio was female biased.

All insects collected in the field showed a body fat rank lower than ranks observed in insects just emerged from seeds. In males, the body fat was low and stable over time (from April 28 to June 28) (mean ± SD = 0.91 ± 0.40; linear regression, *r* = 0.20, R-MSE = 0.19, *n* = 28, *p* = 0.29). In contrast, females were fatter than males, but their body fat decreased over time (mean ± SD = 2.13 ± 0.72; Mann-Whitney, *p* < 0.0001; linear regression, *r* = −0.57, R-MSE = 0.65, *n* = 47, *p* < 0.0001). From the first to the last week of infestation, the rank of body fat in females had decreased from 3 to 0.5.

### Spring-Sown Field: Vegetation Phase

From April 14 to May 8, spring-sown field beans were in a vegetative phase with no flowers at all. No broad bean weevil was observed, while the insects were present on the adjacent winter-sown field beans.

### Spring-Sown Broad Beans: First Week of the Blooming Phase

In spring-sown field beans, the blooming period occurred 2 weeks later than in winter-sown field beans (Figure 4B). The first stage of flowers was observed on May 11, and the first insects were captured in the field on May 6 (Figure 4D). During the first week of infestation, the sex ratio was female biased. Sixty-six percent of males were sexually active and females showed a high ovarian development (ranking between 2 and 4). Seventy-five percent of females had full filled spermatheca.

### Spring-Sown Field: Blooming Phase and Fructification Phase

A high number of inflorescences was observed from May 20. Throughout the monitoring period (from May 6 to June 30), no significant sex ratio bias was observed, 8.12 males (SD ± 8.92) and 6.24 females (SD ± 5.08) were collected (Mann-Whitney's test, *p* = 0.80) (Figure 4B).

At the beginning of the crop colonization, the ovarian development was maximum (rank 4); therefore, the rank did not evolve from May 6 to June 30.

Throughout the same period, the number of mature oocytes showed a peak on May 21 (±0,1 days) (non-linear regression, *R*^2^ = 0.60, R-MSE = 0.23, *n* = 10, Df = 7; Figure 5).

As done for winter-sown field beans, mating in spring-sown field beans was observed in the field, as the number of mated females increased over time. The rate reached a plateau from May 23 (non-linear regression, *R*^2^ = 0.60, R-MSE = 0.23, *n* = 10, Df = 7; Figure 5).

In spring-sown field beans, first pod set was observed on May 25, reaching to a maximal value during June (around 8–12 pods per stem). At that time, few mature oocytes were present in females. A rapid expansion of pods was observed between May 25 and June 1. The number of pods followed an asymptotic curve, reaching a plateau on June 30 with 10.72 ± 0.66 pods. As observed in winter-sown field beans, sex ratio was female biased.

As in winter-sown field beans, females collected from the spring-sown field beans showed a higher rank of body fat (1.95 ± 0.72; mean ± SD) than males (0.86 ± 0.23; mean ± SD; Mann-Whitney, *p* < 0.0001).

### Winter-Sown Field Beans vs. Spring-Sown Field Beans

From April 28 to June 30, 977 beetles were collected (in winter-sown and spring-sown field beans). Density of weevils (per 100 plants observed) was higher in winter-sown field beans (17.55 ± 15.31; mean ± SD) than in spring-sown field beans (10.85 ± 13.67; mean ± SD; Mann-Whitney tests, *p* = 0.03). In both cases, weevils settled down on the crop during the blooming phase. Between the two cultivars, if 15 days separated the blooming of the first flowers, and 19 days the arrival of the first pods, the end of the pod expansion occurred at the same time. This meant that in spring-sown field, the number of pods increased rapidly. As shown through dissection, during the pod expansion time of spring-sown fields, the females had already laid most of their eggs. Contrarily to the spring-sown field, in the winter-sown field, pods came early and over a longer period, allowing a larger period of oviposition time for females.

Throughout the monitoring period, dissection showed that the amount of body fat observed in the males and females collected, had decreased over time (linear regression, male, *r* = −0.67, R-MSE = 0.18, *n* = 11, *p* = 0.02; females, *r* = −0.68, R-MSE = 0.53, *n* = 23, *p* = 0.0003). Between the first and the last week of infestation, the rank of body fat had fallen from 2 to 0.5 in females.

**Figure 6:**
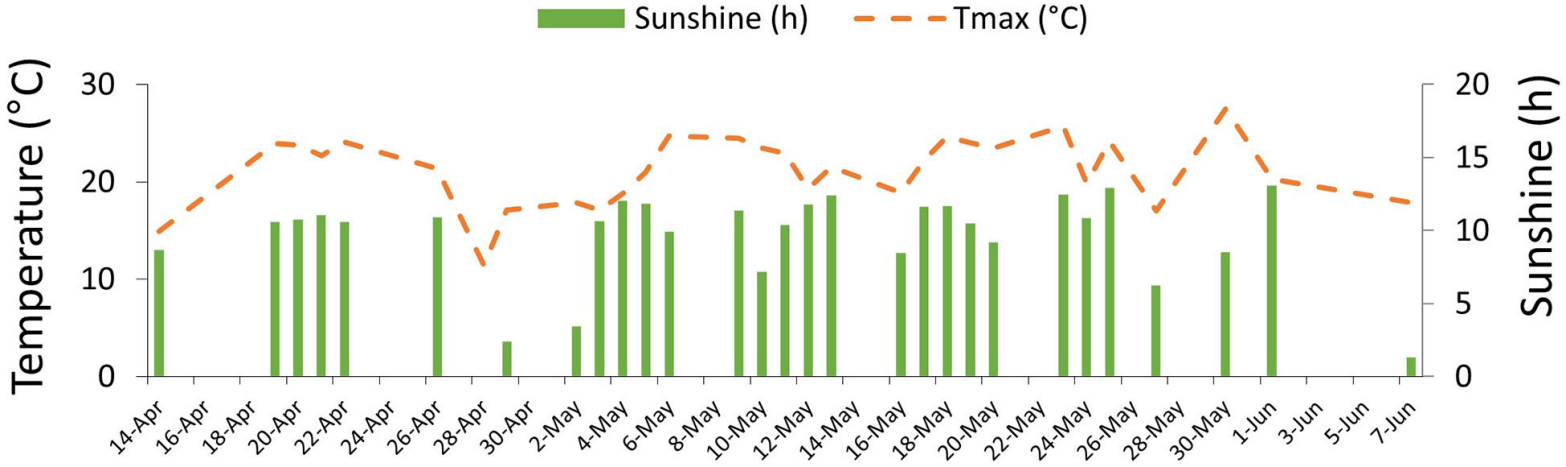
Climatic data recorded from fields.

During the field monitoring, a roughly constant temperature above 15°C was recorded (Tmax means ± SD = 21.93 ± 4.21; Figure 6).

### Ovarian Development and Fat Body Rank in Insects Just Emerged From Seeds

In the study, all adults having emerged from *V. faba* seeds were in reproductive diapause. In females, the ovarioles were reduced to the *germarium* structure (rank 0) and dissection of males showed that the lateral gland and the median gland were not developed. Both sexes showed high ranks of body fat (rank 4 and rank 5, respectively). These high ranks had never been reached in collected field insects.

### Nutrition

All insects captured from winter-sown and spring-sown field beans showed a feeding activity demonstrated by the presence of food residues in the midgut and the hindgut. Dissection showed a large amount of pollen from *V. faba*. Indeed, broad bean weevils on the field, were frequently observed in the inflorescences, feeding on *V. faba* pollen, but also on extrafloral nectaries produced by the black spots of clasping leaves.

**Figure 7:**
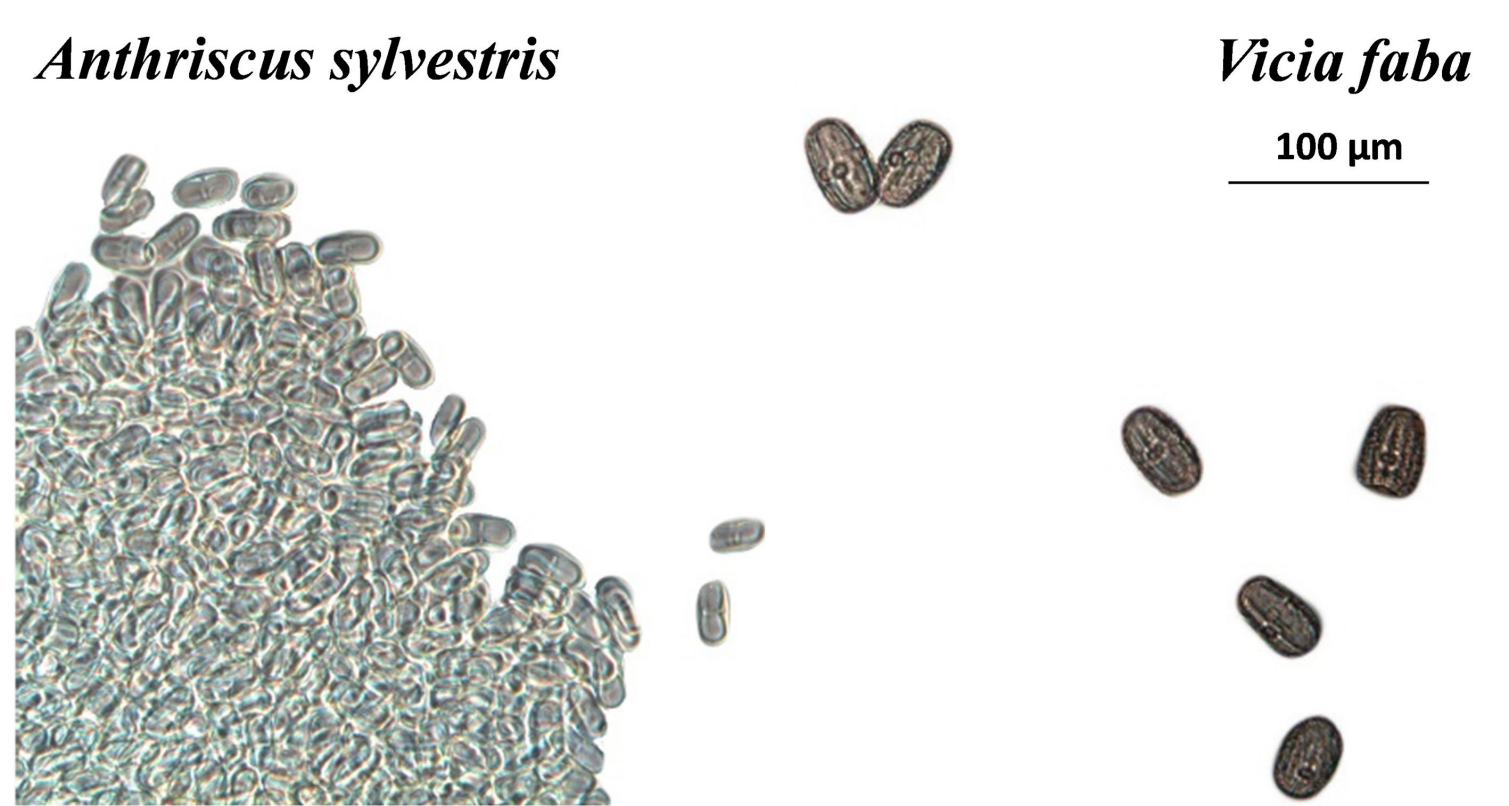
Pollen grains of *Anthriscus sylvestris* and *Vicia faba* found in feces (X50 magnification).

Under artificial conditions, and in all cases, pollen grains were found in the digestive tracts and in the feces of both sexes. By seed pollen comparisons, pollen from *V. faba*, wild chervil, oilseed rape and wild Prunus were found in the feces and guts (Figure 7).

## Discussion

The aims of the study were to answer two questions: (1) Which alternative pollen resources exists for weevils during the vegetative phase of broad bean fields? And (2), could changing sowing dates (i.e., cultivars) lead to modifying their density, and their using the plant resources (i.e., flowers and pods) differently?

Results showed that during the plant vegetative phase, regardless of the cultivar, no broad weevils were on the crops. At that time, the plant did not provide pollen resources. As shown in the present study, oilseed rape and wild plants, such as wild chervil and Prunus, could be used as pollen resources around overwintering sites and fields. It was already known that *B. rufimanus* was not fully exclusive to crops. Adults could reproduce on disparate wild Fabaceae, mainly, the widespread species *Vicia pannonica* (20, 21). For feeding, post overwintering weevils might be temporarily attracted by disparate floral scents emitted by wild plants. Then, the large amount of floral scent from blooming field beans might trigger population movements over a great distance to colonize fields. The present study corroborated the assumption that the first colonizing weevil was linked to the first flowers blooming. Frérot and Leppik (22) have identified different classes of volatile organic compounds emitted by flowers and pods that were involved in the interaction of *B. rufimanus* with the host plants [overviewed in Segers et al. (23)].

Contrarily to wild plants, early blooming phases of winter-sown field provided huge food resources, for post overwintering weevils, and also a stimulating effect on reproduction. The results showed that the sexual maturity of females increased along with the density of flowers, leading to ovipositioning on first pods sets. The results corroborated the laboratory observations of Huignard et al. (4). Thus, for weevils, flowers were a meeting place for mating, which could explain the lack of sexual pheromones (Frérot, personal communication).

Males arrived first to the blooming winter-sown field and were the pioneer sex. Then females came. Whether males produced a sex or an aggregation pheromone to attract female and male congeners is not known (24–27). Bruce et al. (27) identified a sex pheromone, but there were no further reports on the effect of the identified compound. When both cultivars were close from each other, Bruchids migrated from winter-sown to spring-sown field beans, as soon as the flowers appeared in spring-sown field. Doubtless, the flower stage was a positive signal for Bruchids.

The main finding of the study was the evidence of a clear phenological time lag between both cultivars. Early sown beans had an early and longer flower set up and the pod stages lasted longer. Consequently, in the spring-sown field: (1) fewer weevils were present, and (2) at the beginning of the pod set up, females in that field bore few eggs. The study highlighted that the delayed blossoming and pod forming in spring-sown field induced a restricted oviposition. As a consequence, less damage should be observed in spring-sown field than in winter-sown field. Unfortunately, in the present study, damage was not recorded at harvest time. Nevertheless, in Poland and the UK, studies showed that late sowing had led to less seed damaged than early sowing (5, 28), results which echoed those of the present study.

Finally, at the end of pod expansion, the number of weevils had decreased, whatever the sowing dates, but more females than males remained present. Considering the low-fat reserve and the low rate of mature ovocytes in females at that time, it was unlikely that adults were able to move to new places.

## Conclusion

In *B. rufimanus*, the blooming phase of *V. faba* is most likely essential to field location and mating. A perfect synchrony links the weevil to *V. faba*. Flowers provide food resources and sexual stimulations, and pods offer oviposition sites. However, *V. faba* sowing dates affect *B. rufimanus* density and the biological synchrony of females to the pods. Early bloom and long-lasting pod expansion focus the pest to winter-sown field. Pending the blooming time, population of weevils can stay around fields by feeding on wild plants. Controlling surrounding wild plants and developing late pod expansion cultivars may help to reduce damage to bean seeds. Finally, given the high number of adults in winter-sown field, early sowing cultivars could be used in IPM strategies such as trap-cropping or attracting and insect-killing techniques (i.e., lure and kill) (23, 29, 30). The density of adults and seed damage figures should be used to test the effectiveness of those methods. The insect cycle is perfectly synchronized with the phenological development of its host plant. *B. rufimanus* is highly adaptable to the plant phenology.

## Data Availability Statement

The original contributions presented in the study are included in the article/Supplementary Material, further inquiries can be directed to the corresponding author/s.

## Author Contributions

RH: conceptualization, formal analysis, investigation, methodology, and writing (original draft). PT: project administration, funding acquisition, and resources. BF: conceptualization, investigation, validation, methodology, funding acquisition, and writing (review and editing). All authors contributed to the article and approved the submitted version.

## Conflict of Interest

The authors declare that the research was conducted in the absence of any commercial or financial relationships that could be construed as a potential conflict of interest.

## Publisher's Note

All claims expressed in this article are solely those of the authors and do not necessarily represent those of their affiliated organizations, or those of the publisher, the editors and the reviewers. Any product that may be evaluated in this article, or claim that may be made by its manufacturer, is not guaranteed or endorsed by the publisher.
