# Supplementary figures and images for: Physiological Synchrony of the Broad Bean Weevil, Bruchus rufimanus Boh., to the Host Plant Phenology, Vicia faba L
Source: Front Insect Sci. 2021 Aug 6;1:707323. doi: 10.3389/finsc.2021.707323 (PMC10926550; doi:10.3389/finsc.2021.707323)

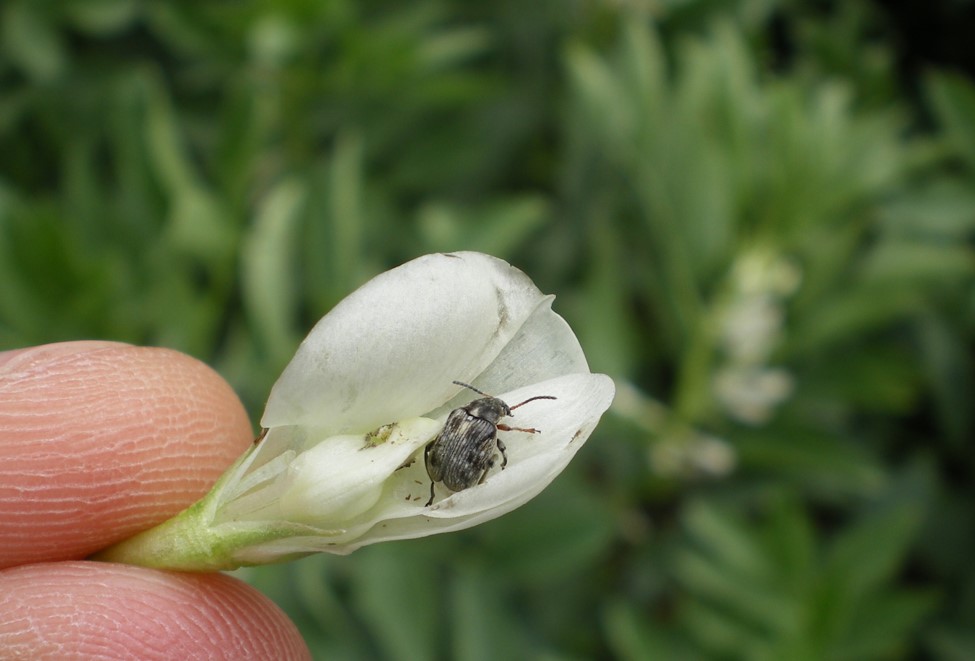

Supplement: Supplementary file 3 [file Image_1.JPEG]
